# Peripheral Reduction of FGFR4 with Antisense Oligonucleotides Increases Metabolic Rate and Lowers Adiposity in Diet-Induced Obese Mice

**DOI:** 10.1371/journal.pone.0066923

**Published:** 2013-07-29

**Authors:** Xing Xian Yu, Lynnetta M. Watts, Vara Prasad Manchem, Kaushik Chakravarty, Brett P. Monia, Michael L. McCaleb, Sanjay Bhanot

**Affiliations:** 1 Department of Antisense Drug Discovery, Isis Pharmaceuticals Inc., Carlsbad, California, United States of America; 2 Department of Clinical Development, Isis Pharmaceuticals Inc., Carlsbad, California, United States of America; Sapienza University of Rome, Italy

## Abstract

Obesity is a primary risk factor for multiple metabolic disorders. Many drugs for the treatment of obesity, which mainly act through CNS as appetite suppressants, have failed during development or been removed from the market due to unacceptable adverse effects. Thus, there are very few efficacious drugs available and remains a great unmet medical need for anti-obesity drugs that increase energy expenditure by acting on peripheral tissues without severe side effects. Here, we report a novel approach involving antisense inhibition of fibroblast growth factor receptor 4 (FGFR4) in peripheral tissues. Treatment of diet-induce obese (DIO) mice with FGFR4 antisense oligonucleotides (ASO) specifically reduced liver FGFR4 expression that not only resulted in decrease in body weight (BW) and adiposity in free-feeding conditions, but also lowered BW and adiposity under caloric restriction. In addition, combination treatment with FGFR4 ASO and rimonabant showed additive reduction in BW and adiposity. FGFR4 ASO treatment increased basal metabolic rate during free-feeding conditions and, more importantly, prevented adaptive decreases of metabolic rate induced by caloric restriction. The treatment increased fatty acid oxidation while decreased lipogenesis in both liver and fat. Mechanistic studies indicated that anti-obesity effect of FGFR4 ASO was mediated at least in part through an induction of plasma FGF15 level resulted from reduction of hepatic FGFR4 expression. The anti-obesity effect was accompanied by improvement in plasma glycemia, whole body insulin sensitivity, plasma lipid levels and liver steatosis. Therefore, FGFR4 could be a potential novel target and antisense reduction of hepatic FGFR4 expression could be an efficacious therapy as an adjunct to diet restriction or to an appetite suppressant for the treatment of obesity and related metabolic disorders.

## Introduction

Obesity has become a worldwide epidemic, especially in the developed countries such as the United States, where about 36% of the adult population and 17% of children and adolescents are obese (http://www.cdc.gov/nchs/data/databriefs/db82.pdf). Obesity is tightly associated with multiple co-morbidities including insulin resistance, type 2 diabetes mellitus, dyslipidemia, hypertension and cardiovascular diseases, which could be improved by reduction of adiposity [1]. The first line treatment for obesity is a regimen of diet and exercise; however, weight regain is the usual outcome after only a few years [2], [3]. A major component to the eventual regain of body weight is the decline in energy expenditure (EE) induced by weight loss [4]–[6]. Thus, a therapeutic strategy that increases EE, or most importantly, maintains the basal EE during a caloric deficit could have substantial efficacy. Since multiple anti-obesity drugs have failed during different stages of clinical development due to unacceptable adverse effects in CNS or due to cardiovascular adverse effects, there remains a great unmet medical need for anti-obesity drugs that increase EE by acting on peripheral tissues without causing cardiac or CNS related side effects [7], [8]. As a class, antisense oligonucleotides (ASO) distribute well to metabolically active tissues such as liver and adipose tissue after systemic administration, but do not distribute well into heart and do not penetrate the blood brain barrier (Dean et al. 2001). Therefore, using ASOs to target genes that regulate metabolism may provide a therapeutic opportunity to increase peripheral energy expenditure without causing CNS or cardiac side-effects. Here we report that treatment of diet-induced obese (DIO) mice with fibroblast growth factor receptor 4 (FGFR4) ASOs showed a significant anti-obesity effect and other metabolic improvements.

FGFR4 is one of the FGFR family members. It is highly expressed in liver (hepatocytes) and some other peripheral tissues, but very lowly expressed in adipose tissue and not expressed in heart tissue. FGFR4 and its ligand, fibroblast growth factor (FGF15) in rodents or its ortholog FGF19 in primates and humans, regulate hepatic bile acid metabolism by regulating the expression of cholesterol 7 alpha-hydroxylase (Cyp7α) and its activity. FGF15/19 is mainly expressed in distal small intestine. Bile acids are excreted into the small intestine during feeding via gallbladder contraction. Increased bile acid concentration in distal small intestine, especially in ileum, in turn induces FGF15/19 expression by binding to the nuclear bile acid receptor FXR [9], which results in an increased FGF15/19 level in circulation. Increasing binding of circulating FGF15/19 to FGFR4 in hepatocytes causes activation of the FGFR4 signaling pathway, consequently reducing bile acid synthesis in liver and refilling of the gallbladder [9]–[11].

In addition to their role in regulation of bile acid metabolism, both FGFR4 and FGF15/19 are also involved in lipid, carbohydrate or energy metabolism. Hepatic FGFR4 expression is decreased by fasting, increased by insulin, and reduced by streptozotocin-induced diabetes [12]. FGFR4 null mice show changed lipid profiles under different nutritional conditions [13], [14]. FGF19 administration to or over-expression in obese mice have been reported to increase metabolic rate, and improve adiposity, liver steatosis, insulin sensitivity and plasma lipid levels [15], [16]. FGF19 was also found to inhibit hepatic fatty acid synthesis [17], to stimulate glycogen synthesis [18], and to decrease hepatic gluconeogenesis [14].

To further investigate the potential metabolic effects of pharmacological inhibition of FGFR4, antisense approach was used to reduce its expression in peripheral tissues in DIO mice. We found that systemic administration of FGFR4 ASO significantly reduced FGFR4 expression in liver, which resulted in reduction of body weight and adiposity, improvement in insulin sensitivity and liver steatosis in DIO mice. This anti-obesity effect of FGFR4 ASO was maintained in caloric restricted animals. When combined with rimonabant, a CNS based appetite-suppressant drug, an additive anti-obesity effect was achieved. Antisense reduction of FGFR4 expression caused increased plasma FGF15 levels, increased tissue fatty acid oxidation rate and whole body metabolic rate, and decreased tissue lipogenesis in these obese mice. No overt toxicities were observed in these studies. These data demonstrate that inhibition of FGFR4 could be a potential therapeutic approach for the treatment of obesity and related metabolic disorders.

## Materials and Methods

### ASOs

Two FGFR4 ASOs that target FGFR4 mRNA in different regions were used in the studies. One FGFR4 ASO was designated as FGFR4 ASO #1 (sequence: 5′-GCCACATTTCCTTCCAGCTG -3′) and the other as FGFR4 ASO #2 (sequence: 5′-TCCATTTCCTCAGAGGCCTC-3′). The chemical composition and mechanism of action of ASOs have been described previously [19], [20]. A control ASO, which has the same chemical composition as FGFR4 ASOs but is not complementary to any known gene sequence, was also used in the studies.

### Animal studies

Studies were conducted in DIO mice. The protocols for the studies were approved by Isis Institutional Animal Care and Use Committee (Permit Numbers: 0198 and 0205). All efforts were made to minimize stressing and suffering. Male, 6–8 week old C57BL/6J mice (Jackson Laboratories, Bar harbor, ME) were housed 3 animals per cage at 22 to 25°C with 12 h light:dark cycle and free access to food and water. The mice were fed a diet containing 58 kcal% fat (Research diet D12330; Research Diets, New Brunswick, NJ) for 2–5 months to induce obesity before the initiation of different treatments. The mice were allocated into different groups based on body weight, body composition and plasma biochemistry (see below for methods), and treated (s.c. injection) with saline, control ASO, or either FGFR4 ASO at different doses, twice a week, for various periods (see Table 1 and related figure legends). Dosages are reported as weekly dose for all the animal studies. For some studies, a group of lean mice fed a regular rodent chow and injected with saline was included as a comparator. For caloric restriction study, caloric restriction was started 2 weeks after the initiation of ASO treatment and 90% of the amount of food consumed daily by FGFR4 ASO treated group during the first two weeks was provided to all three caloric restricted groups while one saline-treated group was remained on free access to food. For combination treatment study, in addition to ASO treatment, mice were given daily oral administration of rimonabant (5 mg/kg) or vehicle 2.5 weeks after the initiation of ASO treatment.

**Table 1:** Summary of the design on the pharmacological studies in DIO mice.

Study	FGFR4 ASO	Weekly dose	Treatment duration
Two FGFR4 ASO study	ASO #1, ASO #2	50 mg/kg/week	10 weeks
Dose-response study	ASO #1	25, 50, or 75 mg/kg/week	13 weeks
Caloric restriction study	ASO #2	50 mg/kg/week	ASO treatment: 8 weeks; caloric restriction: week 3 to 8
Combination study	ASO #1	50 mg/kg/week	ASO treatment: 8 weeks; Rimonabant: week 2.5 to 7.5

During the studies, body weight and food intake were measured weekly; body composition was measured at different time points with an Echo MRI system (Echo Medical System, Houston, TX). In some studies, blood was also collected at different time points for the measurement of plasma glucose, insulin, and other plasma chemistry. At the end of the studies, animals were sacrificed; blood was collected; and tissue samples were dissected, weighed, and stored at −80°C for further analysis.

### Biochemical analysis

Plasma insulin was measured with an insulin ELISA kit (ALPCO Diagnostics, Windham, NH). Plasma FGF15 was determined using an enzyme-linked immunosorbent assay developed in house using mouse FGF15 antibody from Santa Cruz Biotechnology, Inc (Santa Cruz, CA). Plasma glucose and other plasma chemistry parameters were measured with a clinical analyzer (Olympus AU400, Olympus American Inc, Melville, NY).

### Metabolic rate measurement

Metabolic rate in the mice was measured using indirect calorimetry (Oxymax System, Columbus Instruments, Columbus, OH) as described [21].

### Insulin tolerance test (ITT)

ITT was conducted in the mice that were treated with FGFR4 ASO for 5.5 weeks. Before ITT, the mice were fasted for 4 hours. A baseline (0-min) tail blood glucose level was measured followed by administration (i.p. injection) of insulin at 0.5 U/kg for ITT. Tail blood glucose was then measured at 30 min intervals for up to 2 hours using a Glucometer (Abbott Laboratories, Bedford, MA).

### Hyperinsulinemic-euglycemic clamp study

DIO mice treated with control ASO or FGFR4 ASO #1 at 50 mg/kg/week for 4 weeks were subjected to hyperinsulinemic-euglycemic clamp study for further determination of glucose metabolism and insulin sensitivity. Specifically, overnight fasted mice were infused with [3-^3^H] glucose (PerkinElmer Life and Analytical Sciences) through jugular vein to assess basal whole body glucose turnover. Hyperinsulinemic-euglycemic clamp was then conducted with a primed and continuous infusion of human insulin (Humulin; Eli Lilly) to raise plasma insulin within a physiological range. Blood samples were collected for the immediate measurement of plasma glucose concentration, and glucose was infused at a variable rate to maintain euglycemia. Insulin-stimulated whole body glucose turnover was estimated with a continuous infusion of [3-^3^H] glucose at 0.1 µCi/min throughout the clamp procedure. To estimate insulin-stimulated glucose uptake in different tissues, 2-deoxy-D-[1-^14^C]glucose (2-[^14^C]-DG, PerkinElmer Life and Analytical Sciences) was administered as a bolus (10 µCi) at 75 min after the start of clamp study. Blood samples were taken before, during, and at the end of clamps for measurement of plasma [^3^H]-glucose, ^3^H_2_O, 2-[^14^C]-DG concentrations. At the end of clamp, mice were sacrificed, and tissues were collected and saved at −80°C until analysis.

### Bile acid pool size analysis and plasma total bile acid measurement

Samples from DIO mice treated with saline, control ASO or FGFR4 ASO #1 at 50 mg/kg/week for 3–4 weeks were used for the determinations of bile acid pool size as previously described [22] and for the measurement of plasma total bile acid levels using a kit from BQ Kits (San Diego, CA).

### Determination of de novo fatty acid synthesis in vivo

DIO mice treated with saline, control ASO or FGFR4 ASO #1 at 50 mg/kg/week for 6 weeks were used for the determination of *in vivo de novo* fatty acid synthesis. Mice were administered (i.p injection) 10 mCi ^3^H_2_O. One hour later, the mice were sacrificed and white adipose tissue was collected for analysis of the amount of ^3^H-labled fatty acids [19], [23]

### Determination of fatty acid oxidation both in vivo and in vitro

DIO mice treated with control ASO or FGFR4 ASO #1 at either 25 or 50 mg/kg/week for 6 weeks were used for *in vivo* fatty acid oxidation study. The mice were fasted overnight and then orally administered [U-^13^C]-palmitate at 1.0 mg/g body weight. Eight hours later, mice were sacrificed, and plasma, liver and white adipose tissues were collected and used for metabolic profiling as previously described for the determination of fatty acid oxidation [24], [25].

Fatty acid oxidation was also determined *in vitro*. Mouse primary hepatocytes were isolated and the cultured for 24 hours. Fatty acid oxidation in the hepatocytes was then determined after overnight culture with or without recombinant FGF19 (0.5 ng/ml) or AICAR (0.5 mM) by measuring the oxidation of [14C]oleate into CO_2_ as described [19], [23], [26], [27].

### FGF19 infusion study

DIO mice were subcutaneously infused with either saline or recombinant FGF19 (R&D System) at 100 ng/kg/day for 7 days with a subcutaneously implanted mini-pump (Alzet mini-pump). To avoid potential FGF19-caused immune-responses, a longer term infusion was not conducted; thus effect of a longer term FGF19 infusion on BW and adiposity was not evaluated. Mice were bled 3 and 7 days after the initiation of infusion and 3 days after the termination of infusion, and plasma FGF19 levels were measured with an FGF19 ELISA kit (R&D System). Metabolic rate in these mice on the day right before initiation of infusion, day 2 though day 7 during the infusion and day 2 and day 3 after infusion was measured with indirect calorimetry as described above.

The study was repeated in DIO mice treated with FGFR4 ASO #1 at 50 mg/kg /week and fed the 58% HF diet mixed with 2% Welchol, a bile acid sequestrant; the latter was used to eliminate FGFR4 reduction caused increase in plasma FGF15 levels. FGF19 Infusion was initiated three weeks after ASO treatment and Welchol-feeding.

### Western immunoblotting analysis

For FGFR4 and Cyp7α1 protein assays, liver homogenates and liver microsome-enriched fraction containing an equal amount of total protein were separated on SDS-PAGE, respectively, under reduced conditions and then transferred onto PVDF membranes. The blots were then incubated with a primary antibody against mouse FGFR4 (R&D System) or monkey Cyp7α1 (the antibody is cross-reactive to mouse Cyp7α1 and was kindly provided by Dr. Paul Dawson at Wake Forest University). Signals were detected by using a HRP-conjugated secondary antibody and developed with ECL detection reagents (Amershan Biosciences). The blot was then stripped and re-probed with a SRB1 antibody (Novus Biologicals) for loading control and the signals were detected as described above.

### Histological analysis

For H&E staining, epididymal white fat samples and liver samples were fixed in 10% buffered formalin and embedded in paraffin wax. For oil-red O staining, liver samples were collected in embedding medium. Multiple adjacent 4-μm sections were cut and mounted on glass slides. After dehydration, the sections were stained. Images of the histological sections were analyzed.

### Gene expression analysis

Total RNA was isolated by homogenizing tissues in RLT buffer (Qiagen, Maryland) followed by centrifugation with cesium chloride gradient. Real-time quantitative RT-PCR analysis was then performed using sequence-specific primers and probes to analyze the gene expression as described [19], [20].

### Statistical analysis

Values presented represent the mean ± SEM. Statistical difference between treatment groups was determined using one-way ANOVA with Tukey HSD multiple comparisons or two-tailed student t-test. *P*<0.05 was considered to be significant.

## Results

### FGFR4 ASO reduced liver FGFR4 expression and lowered adiposity in DIO mice fed *ad libitum*

**Figure 1:**
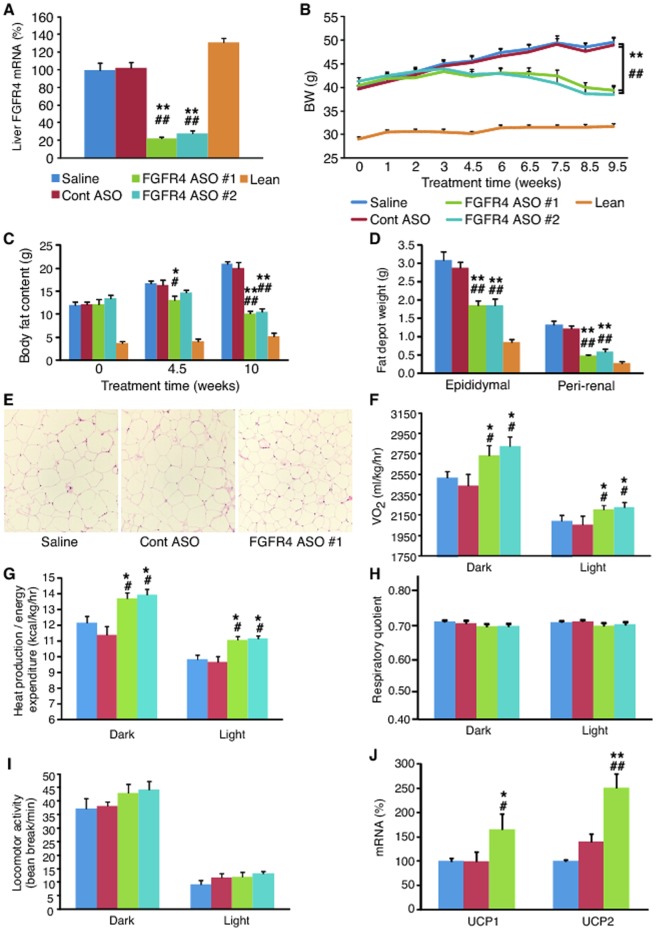
FGFR4 ASO reduced hepatic FGFR4 gene expression, and lowered adiposity in DIO mice fed *ad libitum*. DIO mice were treated with saline, control (Cont) ASO or FGFR4 ASO #1 or #2 at 50 mg/kg/week for 10 weeks; a group of lean mice treated with saline was used as normal controls. (A) Liver FGFR4 mRNA levels after treatment; measured with quantitative RT-PCR. (B) BW changes during the treatment. (C) Total body fat content at different time points of the treatment, measured with an Echo-MRI body composition analyzer. (D) Epididymal and peri-renal fat depot weights at the end of the treatment. (E) H&E staining of epididymal fat tissue harvested at the end of the treatment. (F) Whole body O_2_ consumption rate (VO_2_), (G) whole body heat production rate (energy expenditure rate), (H) respiratory quotient and (I) locomotor activity measured with an indirect calorimetry. (J) UCP1 and UCP2 mRNA levels in brown adipose tissue of DIO mice after treatment with saline, Cont ASO or FGFR4 ASO #1 at 50 mg/kg/week for 10 week. Data are expressed as mean ± SEM (n = 6–9/group). *P<0.05 and **P<0.01 vs. saline controls; ^#^ *P*<0.05 and ^##^ *P*<0.01 vs. Cont ASO.

To investigate the metabolic effects of pharmacological reduction of FGFR4 expression on body weight and adiposity, antisense approach was used to treat DIO mice fed ad libitum. To make sure that the effects caused were specific to the reduction of the target expression, the mice were treated with two different FGFR4 ASOs, FGFR4 ASO #1 or FGFR4 ASO #2, which target FGFR4 mRNA at different regions. After 10 weeks of treatment at a dose of 50 mg/kg/week, both FGFR4 ASOs reduced liver FGFR4 gene expression by >70% (P<0.01; Figure 1A) as compared to saline controls whereas control ASO treatment did not change its expression.

Treatment with either FGFR4 ASO lowered body weight (BW) of the obese mice by >20% whereas control ASO did not change BW as compared to saline controls (Figure 1B). Lower BW was observed in FGFR4 ASO-treated groups within 4.5 weeks of treatment. The reduction of BW by FGFR4 ASO treatment was due to a decrease in body fat content (Figure 1C) because the treatment caused no changes in lean body mass (25.5±0.9 g in saline; 24.5±0.8 g in FGFR4 ASO #1; 23.9±1.0 g FGFR4 ASO #2 at sacrifice). A decrease of body fat content was also reflected as marked reductions in epididymal and peri-renal fat depot weights (Figure 1D). Histological examination with H&E staining showed a reduced size of adipocytes in adipose tissue of FGFR4 ASO-treated mice (Figure 1E) with no redistribution of triglycerides into either liver or muscle. In fact, liver histological examination demonstrated an improvement in liver steatosis (see below). Neither FGFR4 ASO caused significant change in food intake, indicating that improved adiposity was caused by an increased metabolic rate, which was confirmed by indirect calorimetry measurement showing that both FGFR4 ASO groups had higher O_2_ consumption rate (VO_2_) and heat production rate as compared to controls (Figure 1F and 1G). A trend of decrease in respiratory quotient was also observed in both FGFR4 ASO-treated groups (Figure 1H). The high-fat diet feeding resulted in a very low respiratory quotient (approximately 0.7 in controls), which could limit it to be further significantly lowered by the treatment. Neither FGFR4 ASO caused significant change in locomotor activity (Figure 1I), indicating that the increased VO_2_ and heat production were caused by an increased basal metabolic rate. Gene expression analysis on the samples from another study in which DIO mice showed similar magnitudes of reduction in both BW and adiposity after FGFR4 ASO #1 treatment at 50 mg/kg/week for 10 weeks found that the treatment increased UCP1 expression by 1.7-fold and UCP2 expression by 2.5-fold in brown adipose tissue (Figure 1J).

**Figure 2:**
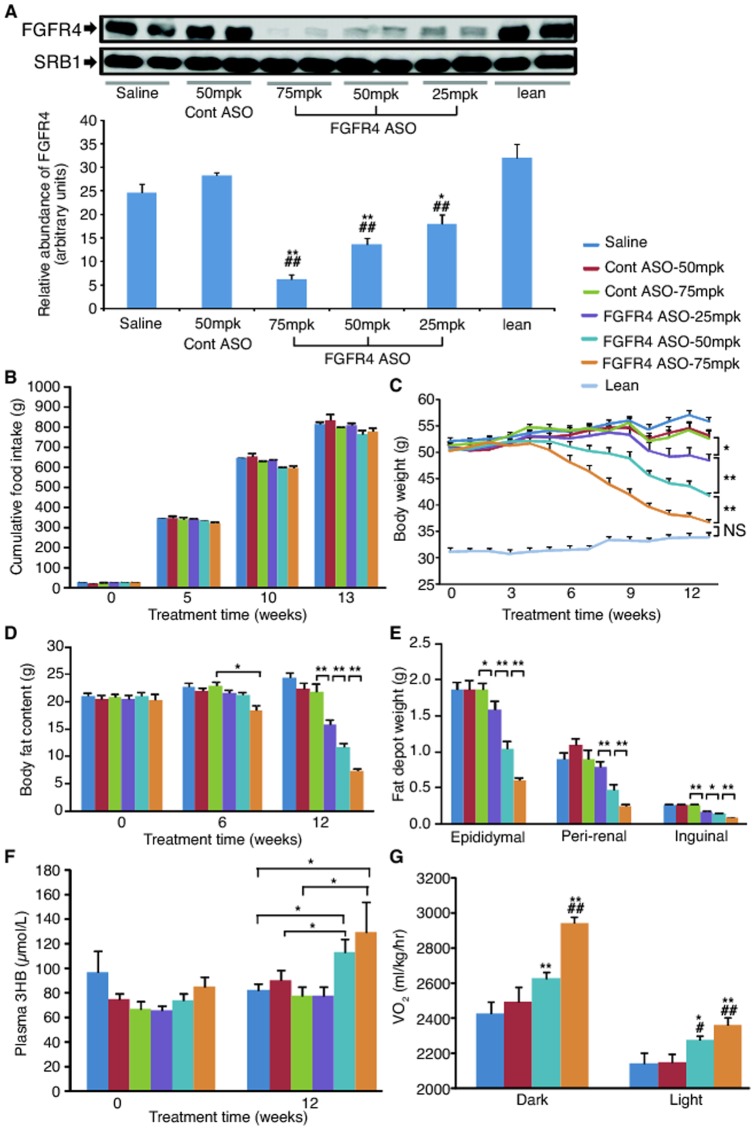
FGFR4 ASO dose-dependently reduced FGFR4 expression, and lowered adiposity and increased whole body metabolic rate. DIO mice were treated with saline, control (Cont) ASO or FGFR4 ASO #1 at 25, 50, or 75 mg/kg/week for 13 weeks; a group of lean mice treated with saline was used as normal controls. (A) Upper panel, representative Western blots of liver FGFR4 protein and SRB1 protein (as loading control); lower panel, the quantitative values of liver FGFR4 protein after normalizing to SRB1. (B) Cumulative food intake during the treatment. (C) BW changes during the treatment. (D) Total body fat content at different time points of the treatment. (E) Fat depot weights at the end of the treatment. (F) Plasma β-hydroxybutyrate (3HB) levels at week 0 and week 12. (G) Whole body O_2_ consumption rate (VO_2_). Data are expressed as mean ± SEM (n = 6–9/group). *P<0.05 and **P<0.01 vs. saline controls, or as indicated in the figure; ^#^ *P*<0.05 and ^##^ *P*<0.01 vs. Cont ASO; NS, not significant.

To evaluate the dose dependency of the anti-obesity effect induced by antisense reduction of FGFR4 expression, DIO mice were treated with FGFR4 ASO #1 at 25, 50 and 75 mg/kg/week for 13 weeks. Liver FGFR4 mRNA was reduced by 35%, 55% and 62%, respectively; and liver FGFR4 protein level was also reduced dose-dependently with approximately a 75% reduction at the highest dose as compared to the saline control, whereas it was not changed with control ASO treatment (Figure 2A). Again, treatment with FGFR4 ASO did not cause significant decreases in food intake (Figure 2B) during the entire period of the treatment, but it caused a dose-dependent and time-dependent reduction of both BW (Figure 2C) and total body fat content (Figure 2D). Remarkably, at the end of the study, BW was reduced by 34% (P<0.01) and total body fat content was reduced by 70% (P<0.01) at the highest dose as compared to saline controls. As expected, the treatment resulted in a dose-dependent reduction in the weight of epididymal, peri-renal and inguinal fat depots (Figure 2E), indicating an improvement in both visceral and subcutaneous adiposity. In addition, the higher doses of FGFR4 ASO lowered plasma triglycerides levels (by 27% at 50 mg/kg/week and 29% at 75 mg/kg/week) and total cholesterol levels (by 19% at 75 mg/kg/week). Furthermore, the higher doses increased plasma β-hydroxybutyrate (3HB) levels (Figure 2F), suggesting increased hepatic fatty acid oxidation. Indirect calorimetry measurement showed that FGFR4 ASO dose-dependently increased VO_2_ in both dark and light phases (Figure 2G) without changing locomotor activity as compared to controls. These data further demonstrate that antisense reduction of FGFR4 expression caused upregulation of whole body basal metabolic rate, which resulted in improvement of adiposity.

### FGFR4 ASO treatment lowered body weight and improved adiposity in DIO mice under caloric restriction

**Figure 3:**
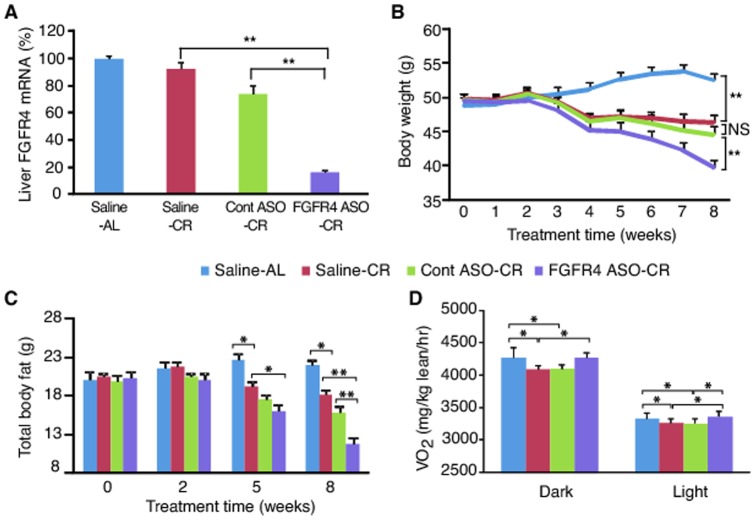
FGFR4 ASO reduced hepatic FGFR4 expression, and lowered adiposity of DIO mice under caloric restriction. DIO mice were treated with saline, control (Cont) ASO or FGFR4 ASO #2 at 50 mg/kg/week for 8 weeks. Two weeks after the initiation of the treatment, Cont ASO-treated group (Cont ASO-CR), FGFR4 ASO-treated group (FGFR4 ASO-CR) and a group of saline-treated mice (Saline-CR) were subjected to caloric restriction by providing 90% of the amount of the food consumed daily by FGFR4 ASO group during the first 2 weeks of treatment; another group of saline-treated mice (Saline-AL) remained on free access to food. (A) Liver FGFR4 mRNA levels after 8 weeks of treatment. (B) BW changes during the treatment. (C) Total body fat content at different time points of the treatment. (D) Whole body O_2_ consumption rate (VO_2_). Data are expressed as mean ± SEM (n = 6–9/group). *P<0.05; **P<0.01; NS, not significant.

To investigate whether reduction of FGFR4 would prevent the adaptive reduction of metabolic rate during caloric restriction, and hence further lower adiposity, DIO mice were treated with FGFR4 ASO #2 in combination with 10% reduction of food intake. Caloric restriction did not cause significant change in liver FGFR4 gene expression (Figure 3A). FGFR4 ASO reduced liver FGFR4 gene expression by 80% (Figure 3A). As expected, caloric restriction lowered both BW (Figure 3B) and body fat content (Figure 3C) in both saline- and control ASO-treated mice as compared to saline-treated mice fed *ad libitum*. Interestingly, treatment with FGFR4 ASO further reduced both BW (Figure 3B) and total body fat content (Figure 3C) in a time-dependent manner. At the end of the study, BW and body fat content were approximately 15% (P<0.01) and 35% (P<0.01) lower, respectively, in FGFR4 ASO-treated mice versus saline-treated, calorie-restricted controls (Figure 3B and 3C). Calorie-restricted mice had reduced VO_2_ as predicted (Figure 3D). Treatment with FGFR4 ASO did prevent the caloric restriction-induced reduction in O_2_ consumption during both dark phase and light phase (Figure 3D), indicating that the decreased adiposity in FGFR4 ASO-treated mice was due to a higher metabolic rate.

### Combination treatment with FGFR4 ASO and CB1 receptor antagonist caused greater reduction of adiposity

**Figure 4:**
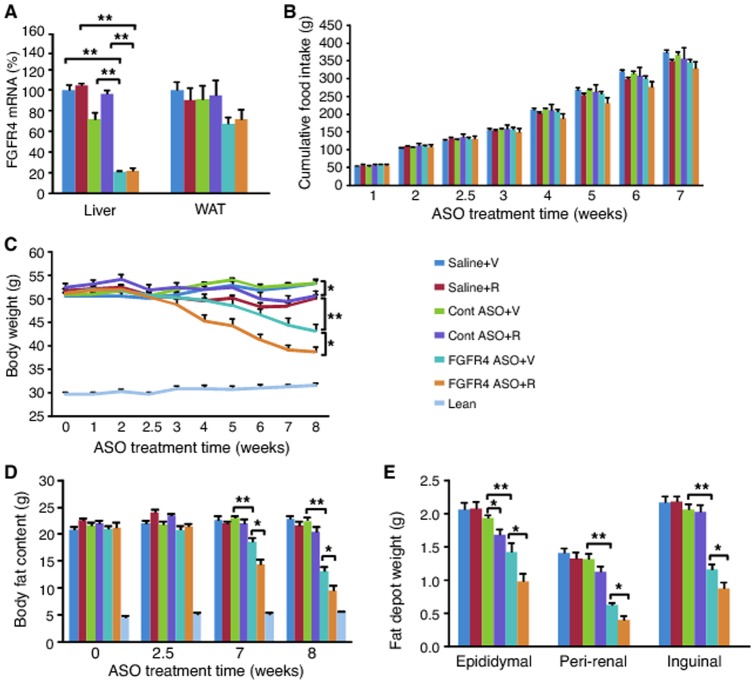
Combination treatment with FGFR4 ASO and rimonabant showed additive reduction of BW and adiposity. DIO mice were treated with saline, control ASO or FGFR4 ASO #1 at 50 mg/kg/week for 8 weeks. Two and half weeks after the initiation of the treatment, the mice were given daily oral administration of rimonabant (R) at 5 mg/kg or vehicle (V) for 5 weeks. (A) FGFR4 mRNA levels in liver and white adipose tissue (WAT) after 8 weeks of the treatment. (B) Cumulative food intake during the treatment. (C) BW changes during the treatment. (D) Total body fat content at different time points of the treatment. (E) Fat depot weights at the end of the treatment. Data are expressed as mean ± SEM (n = 9/group). *P<0.05; **P<0.01.

To investigate whether simultaneous inhibition of FGFR4 and CB1 receptors causes a greater anti-obesity effect, DIO mice were treated with FGFR4 ASO, control ASO or saline for 8 weeks. Two and an half weeks after ASO treatment, the mice received additional treatment with either rimonabant, a CB1 receptor inhibitor, or vehicle for 5 weeks. FGFR4 ASO reduced liver FGFR4 ASO by approximately 80% in either presence or absence of rimonabant (Figure 4A). Addition of rimonabant to the treatments caused some reduction in food intake (Figure 4B). At the end of the study, both saline plus rimonabant and control ASO plus rimonabant groups had lower BW than saline plus vehicle group and control ASO plus vehicle group, respectively (Figure 4C). Treatment with FGFR4 ASO alone caused 19% reduction in BW (Figure 4C) and 42% reduction in total body fat content (Figure 4D) as compared to saline controls. Treatment with FGFR4 ASO plus rimonabant, however, caused as much as a 27% reduction in BW (Figure 4C) and 58% reduction in total body fat content (Figure 4D). Similarly, treatment with FGFR4 ASO alone caused 31%, 55% and 47% reduction in epididymal, peri-renal and inguinal fat depot weights, respectively, as compared to saline controls (Figure 4E). Combination treatment with FGFR4 ASO and rimonabant caused a greater reduction in all these fat depots (Figure 4E). Therefore, these data demonstrate that simultaneous inhibition of FGFR4 and CB1 receptors caused an additive anti-obesity effect in DIO mice.

### FGFR4 ASO treatment increased in vivo fatty acid oxidation and decreased fatty acid synthesis

**Figure 5:**
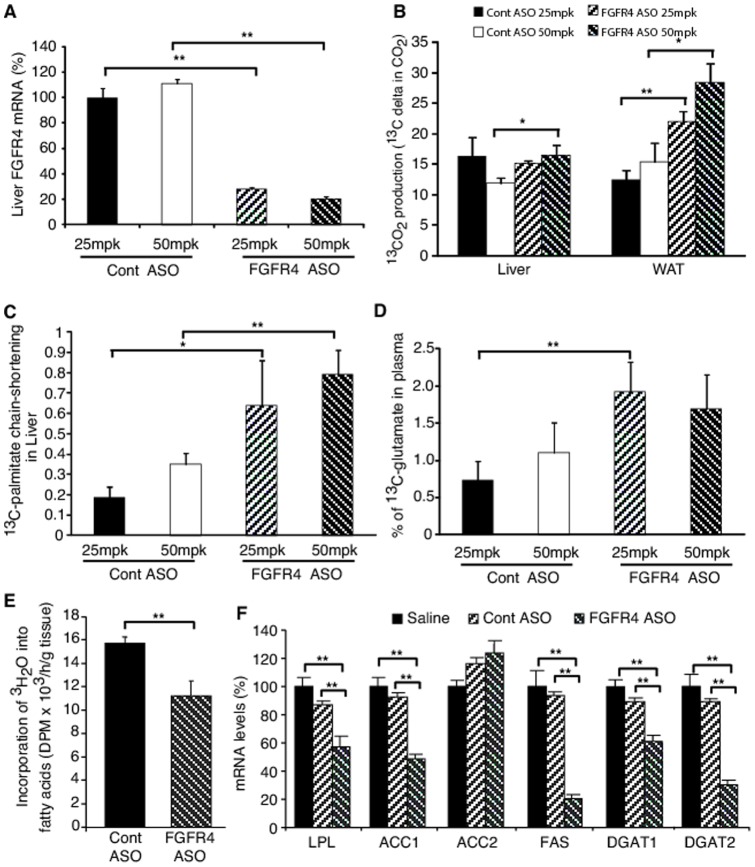
FGFR4 ASO treatment increased fatty acid oxidation and decreased *de novo* fatty acid synthesis. DIO mice treated with control ASO or FGFR4 ASO #1 at 25 or 50 mg/kg/week for 6 weeks were fasted overnight and then orally administered with [U-^13^C]-palmitate at 1.0 mg/g for determination of fatty acid oxidation through metabolic profiling as previously described [24], [25]. Another set of the high-dose ASO treated mice were administered (i.p injection) 10 mCi ^3^H_2_O for determination of *de novo* fatty acid synthesis in adipose tissue or sacrificed directly for the measurement of lipogenic gene expression. (A) Liver FGFR4 mRNA levels after treatment. (B) ^13^CO_2_ production from [U-^13^C]-palmitate oxidation derived from liver and white adipose tissue (WAT). (C) Relative rate of [U-^13^C]-palmitate chain-shortening in liver. (D) Percent ^13^C-glutamate in plasma derived from tissue [U-^13^C]-palmitate oxidation. (E) The incorporation rate of ^3^H_2_O into fatty acids in WAT. (F) Lipogenic gene expression in WAT. Data are expressed as mean ± SEM (n = 6). *P<0.05; **P<0.01.

*In vivo* fatty acid oxidation was determined through metabolic profiling of the plasma and tissues from FGFR4 ASO-treated DIO mice challenged with [U-^13^C]-palmitate. FGFR4 ASO treatment, which dose-dependently reduced liver FGFR4 gene expression (Figure 5A), caused a dose-dependent increase in ^13^CO_2_ production from [U-^13^C]-palmitate oxidation derived from fat tissue (Figure 5B). The high dose treatment with FGFR4 ASO also caused an increase in ^13^CO_2_ production from [U-^13^C]-palmitate oxidation derived from liver (Figure 5B). In addition, there was a dose dependent increase in chain-shortened [U-^13^C]-fatty acid content derived from liver (Figure 5C), suggesting increased β-oxidation of fatty acids in this tissue. These data indicate that treatment with FGFR4 ASO increased *in vivo* fatty acid oxidation in both liver and fat. FGFR4 ASO-treated mice also had increased plasma ^13^C-glutamate levels versus controls (Figure 5D). Plasma ^13^C-glutamate can originate from liver, fat, as well as other tissues. Increased plasma ^13^C-glutamate levels in FGFR4 ASO-treated mice indicated an increase not only in β-oxidation of [U-^13^C]-palmitate, but also in TCA cycle activity coupled with increased conversion of ^13^C-α-ketoglutarate to ^13^C-glutamate in these tissues. Taken together, these data demonstrate that antisense reduction of FGFR4 expression increases fatty acid oxidation in mice.

To investigate whether antisense reduction of FGFR4 expression affects lipogenesis, DIO mice treated with control ASO or FGFR4 ASO were administered ^3^H_2_O to determine the *de novo* fatty acid synthesis in white adipose tissue. In addition, the expression of the key lipogenic genes was also determined after FGFR4 ASO treatment. FGFR4 ASO caused approximately 30% decrease in the incorporation of ^3^H_2_O into fatty acid in the white adipose tissue (Figure 5E), indicating a decreased rate of de novo fatty acid synthesis in this tissue. Consistent with this, FGFR4 ASO treatment caused marked down-regulation of several key lipogenic genes in this tissue, including LPL, ACC1, FAS, DGAT1 and DGAT2 (Figure 5F). FGFR4 ASO treatment also caused down-regulation of the key hepatic lipogenic genes, suggesting decreased lipogenesis in liver as well (see below).

### FGFR4 ASO treatment increased bile acid pool size and plasma FGF15 levels

**Figure 6:**
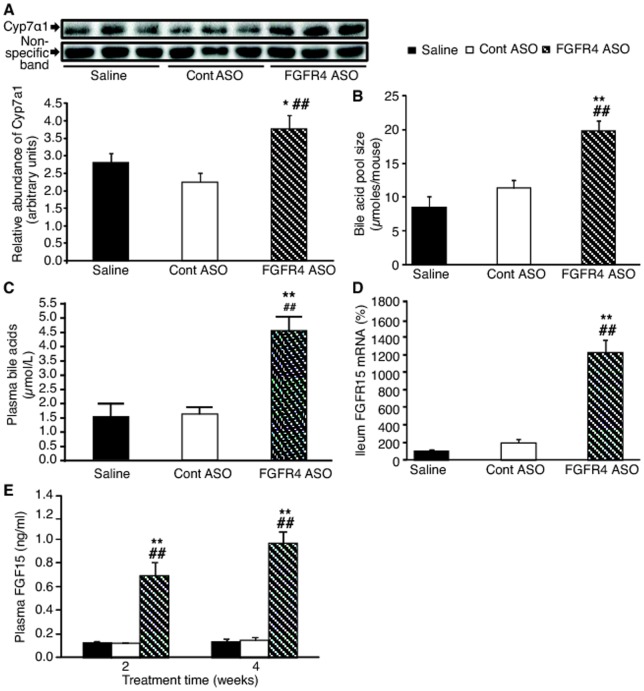
FGFR4 ASO treatment induced Cyp7α1 expression-bile acid synthesis-FGF15 expression pathway. (A) Liver Cyp7α1 protein expression after 4 weeks of treatment with saline, control ASO or FGFR4 ASO #1. Upper panel, representative Western blots of liver Cyp7α1 protein and a non-specific protein band (as loading control); lower panel, the quantitative values of liver Cyp7α1 protein after normalizing to the non-specific protein band. (B) Bile acid pool size, (C) plasma total bile acid levels and (D) ileum FGF15 gene expression after 4 weeks of treatment with saline, control ASO or FGFR4 ASO #1 (n = 5-9/group). (E) Plasma FGF15 levels after 2 and 4 weeks of treatment (n = 9/group). Data are expressed as mean ± SEM. *P<0.05 and **P<0.01 vs. saline controls; ^##^ *P*<0.01 vs. Cont ASO.

To determine the underlying mediator(s) responsible for inducing the anti-obesity effects of FGFR4 reduction, potential changes in the FGF15-bile acid pathway were investigated. FGFR4 ASO treatment caused increase in liver Cyp7α1 protein levels by 35–65% (Figure 6A). It also caused increase in bile acid pool size by approximately 100% (Figure 6B), in plasma total bile acid levels by approximately 200% (Figure 6C) and in ileum FGF15 mRNA expression by 6–12- fold (Figure 6D) as compared to either saline-treated or control ASO-treated mice, which result in significant increase in plasma FGF15 levels (Figure 6E). Actually, plasma FGF15 level was time-dependently increased by FGFR4 ASO treatment with showing approximately 5-fold and 7-fold increases after 2 and 4 weeks of treatment, respectively (Figure 6E).

### Infusion of FGF19 increased metabolic rate in DIO mice treated with or without FGFR4 ASO

**Figure 7:**
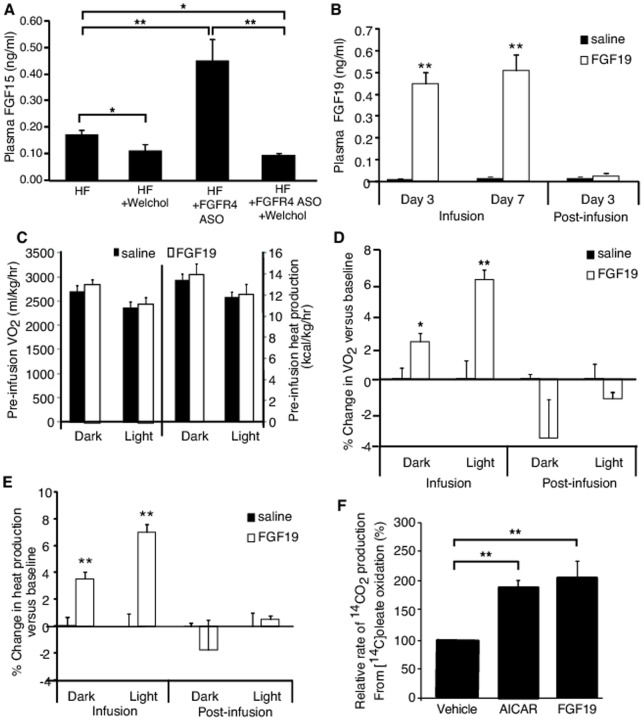
FGF19 increased metabolic rate in vivo and fatty acid oxidation in hepatocytes in vitro. Welchol-feeding reduced plasma FGF15 levels in DIO mice treated with or without FGFR4 ASO (n = 8–9/group; A). Subcutaneous infusion of recombinant FGF19 to mice at 100 ng/kg/day raised the plasma FGF19 levels similar to the FGF15 levels observed in FGFR4 ASO treated mice, which was diminished 3 days post-infusion (n = 8/group; B). Neither whole body VO_2_ nor heart production rate showed difference between two groups before infusion (C). The infusion of FGF19 caused increases in both VO_2_ (D) and heat production rate (E) as compared to the pre-infusion baseline values, which were diminished with the termination of infusion. (F) FGF19 also increased fatty acid oxidation rate in *in vitro* mouse primary hepatocytes when they were treated with vehicle, 1.0 mM AICAR (as a positive control) or 0.5 ng/ml FGF19 (n = 5/group). Data are expressed as mean ± SEM. *P<0.05 and **P<0.01 vs. saline group.

To further determine whether the increased FGF15 level is an underlying mediator, DIO mice treated with or without FGFR4 ASO plus Welchol were infused continually with recombinant, biologically active FGF19 at a dose of 100 ng/kg/day for 7 days. Welchol feeding not only reduced the plasma FGF15 levels in the mice without FGFR4 ASO treatment, but also suppressed the plasma FGF15 levels in FGFR4 ASO-treated mice to the levels of mice without FGFR4 ASO treatment (Figure 7A). The infusion raised the plasma FGF19 levels close to the plasma FGF15 levels seen after FGFR4 ASO treatment in DIO mice (Figure 7B). In addition, the plasma FGF19 levels were minimized 3 days after the termination of the infusion (Figure 7B). Neither VO_2_ nor heat production rate showed difference between two groups before infusion (Figure 7C). FGF19, but not saline, caused significant increases in VO_2_ (Figure 7D) and whole body heat production as well (Figure 7E) during infusion in the mice without FGFR4 ASO treatment or with FGFR4 ASO treatment plus Welchol feeding. The magnitudes of the increases were similar to what were seen in the DIO mice treated with FGFR4 ASO as shown above. These increases disappeared with minimizing of the plasma FGF19 levels after the termination of the infusion (Figure 7D and 7E). In addition, mouse primary hepatocytes cultured with FGF19 at a concentration close to the plasma FGF15 levels found in FGFR4 ASO-treated mice showed an increased fatty acid oxidation rate (Figure 7F), which is in line with the increased fatty acid oxidation in liver and increased 3HB in plasma in FGFR4 ASO-treated mice shown above. Taken together, these data demonstrate that the upregulated FGF15 levels in the DIO mice after FGFR4 ASO treatment is a major mediator for the FGFR4 ASO-induced anti-obesity effect.

### FGFR4 ASO treatment improved insulin sensitivity and liver steatosis

**Figure 8:**
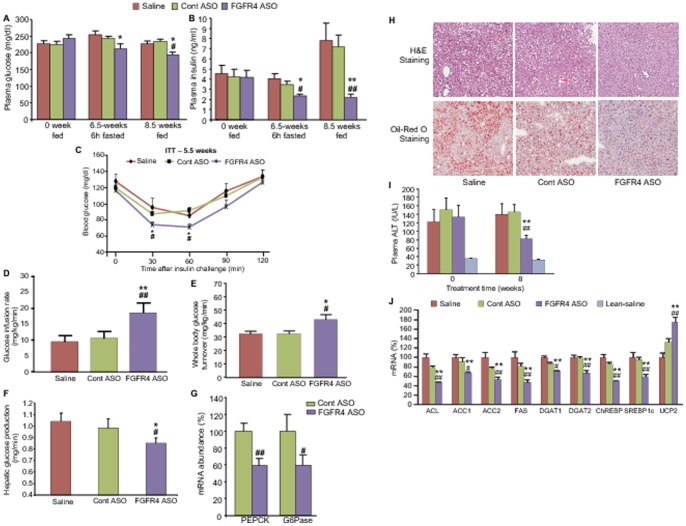
FGFR4 ASO treatment improved insulin sensitivity and liver steatosis in DIO mice. DIO mice treated with saline, control ASO or FGFR4 ASO #1 at 50 mg/kg/week for 6–9 weeks. (A) Plasma glucose and (B) insulin levels at different time points under different nutritional states. (C) Insulin tolerance test (ITT) after 5.5 weeks of treatment. (D) Glucose infusion rate, (E) whole body glucose turnover rate, and (F) hepatic glucose production rate during hyperinsulinemic-euglycemic clamp study. (G) Hepatic gluconeogenic gene expression. (H) Representative pictures of both H&E staining and oil-red O staining showing smaller and fewer fat droplets in the liver from FGFR4 ASO-treated mice. (I) Plasma ALT levels before and after 8 weeks of treatment. (J) Hepatic gene expression after the treatment. Data are expressed as mean ± SEM (n = 6–9/group). *P<0.05 and **P<0.01 vs. saline controls; ^#^ *P*<0.05 and ^##^ *P*<0.01 vs. Cont ASO.

Improvement of glycemia and insulin sensitivity as well as other co-morbidities is also a therapeutic objective of anti-obesity treatments. DIO mice receiving FGFR4 ASO had significantly lower plasma glucose (Figure 8A) and insulin levels (Figure 8B) in both fed and fasted states as compared to controls. After 6 hours of fasting at week 6.5 of treatment, plasma glucose and insulin levels were 16% and 42% lower, respectively, in FGFR4 ASO-treated mice than those in saline controls. At week 8.5, fed plasma glucose and insulin levels were 14% and 69% lower, respectively, in FGFR4 ASO-treated mice than those in saline controls. These data suggest that FGFR4 ASO treatment improved insulin sensitivity. This was confirmed by an improvement of glucose excursion in FGFR4 ASO-treated mice during insulin challenge (Figure 8C). Further confirmation was demonstrated by hyperinsulinemic-euglycemic clamp study, in which FGFR4 ASO-treated DIO mice had an approximate 100% increase in glucose infusion rate (Figure 8D), >30% increase in whole body glucose turnover rate (Figure 8E) and an approximate 20% decrease in hepatic glucose production rate (Figure 8F) as compared to saline controls. Gene expression analysis found that FGFR4 ASO treatment caused down-regulation of hepatic PEPCK and G6Pase, two rate-limiting enzymes for gluconeogenesis (Figure 8G).

In addition, treatment with FGFR4 ASO up to 12 weeks did not cause increase in plasma ALT levels, indicating no liver toxicity after chronic reduction of FGFR4 expression. Rather, the treatment ameliorated liver steatosis and the abnormality of liver function caused by high-fat diet feeding. Liver histological examination with both H&E staining and oil-red O staining demonstrated much smaller and fewer fat droplets in hepatocytes of FGFR4 ASO-treated mice versus controls (Figure 8H), indicating an improvement in liver steatosis. This improvement was accompanied by an improved liver function as reflected by lower plasma ALT levels after FGFR4 ASO treatment (Figure 8I). Gene expression analysis found that FGFR4 ASO treatment caused significant down-regulation of the expression of the key hepatic lipogenic genes, including ATP-citrate lyase (ACL), ACC1, ACC2, FAS, DGAT2, ChREBP, and SREBP1c (Figure 8J). These data, coupled with the data on the hepatic fatty acid oxidation and ketogenesis described above, indicated that improved liver steatosis after FGFR4 ASO treatment was attributable to decreased lipogenesis and increased fatty acid oxidation.

## Discussion

Obesity is caused by a chronic positive energy balance, such that energy intake is more than energy expenditure (EE). As a means to change energy balance, many appetite-suppressing drugs targeting the CNS have been developed for obesity treatment [7], [8], [28]. However, these drugs either failed at different development stages or had to be removed from the market [28] often due to severe side effects, especially the adverse effects in CNS or heart. Alternatively, identification and development of a drug targeting peripheral tissues to increase basal energy metabolism has become a desired approach for the treatment of obesity [29], [30]. In the current studies, we have identified a potential therapeutic approach in which reduction of FGFR4 expression in the periphery with an antisense drug caused a dramatic reduction of the excess adiposity of DIO mice. Furthermore, FGFR4 ASO treatment significantly improved insulin sensitivity, resulting in decreased plasma glucose and insulin levels. FGFR4 ASO treatment also improved liver steatosis and plasma lipid levels. These data indicate that specific inhibition of FGFR4 expression with an antisense drug reduced obesity and improved related metabolic defects in DIO mice.

The anti-obesity efficacy of FGFR4 ASO was primarily due to increased basal EE (coupled with decreased lipogenesis), as supported by the following evidence: First, FGFR4 ASO treatment increased whole body O_2_ consumption and heat production without changing food consumption or locomotor activity. Secondly, the in vivo [U-13C]-palmitate challenge study demonstrated that FGFR4 ASO treatment increased fatty acid oxidation in liver, fat and maybe in other tissues as well (the increased circulating^13^C-glutamate could also be derived from tissues other than fat and liver); the ^3^H_2_O challenge study indicated that FGFR4 ASO treatment decreased de novo fatty acid synthesis in adipose tissue, which was associated with decreased lipogenic gene expression. Lastly, FGFR4 ASO treated mice showed increase of plasma 3HB levels, suggesting increased hepatic fatty acid oxidation in these mice. Thus, the data from the current studies demonstrated that peripheral reduction of FGFR4 with an antisense drug caused an increase of whole body basal EE, thereby reducing adiposity.

Importantly, we found that FGFR4 reduction not only elevated basal metabolic rate during free-feeding conditions, but also prevented its decrease induced by caloric restriction, which resulted in a further loss of adiposity under caloric restriction. Dieting is a primary approach for treating obesity. However, chronic caloric restriction causes an adaptive decrease in metabolic rate, which counteracts the anti-obesity effect of lower energy intake [31] and often results in a poor success rate for long-term weight reduction [2], [3]. Our data indicate that FGFR4 antisense drug has the potential to be combined with diet restriction to provide long-term weight loss therapy.

In the current studies, we also found that antisense reduction of FGFR4 expression coupled with inhibition of CB1 receptor caused an additive effect to reduce BW and body fat content. CB1 receptor is one of the major receptors for endocannabinoid system, which was found to be involved in appetite regulation [32]. Rimonabant is a CB1 receptor inverse agonist and administration of it to both animals [33], [34] and humans [35] has anti-obesity effect, which is believed to be mainly through suppression of food intake. Here, we demonstrated that monotherapy with either rimonabant or FGFR4 ASO reduced BW and body fat content, while combination treatment with both drugs showed additive weight loss. These results indicate that increasing metabolic rate by FGFR4 antisense drug combined with inhibition of caloric intake by an appetite suppressant could be an effective therapeutic approach for the treatment of obesity.

To understand how a reduction of FGFR4 can increase metabolic rate, experiments were performed to identify the primary mediators. In the current studies, we found that antisense reduction of FGFR4 expression caused a multiple-fold induction of plasma FGF15 levels. In addition to its involvement in regulation of bile acid synthesis [9]–[11], FGF15/19 has been found to play an important role in whole body energy metabolism. Transgenic overexpression of FGF19 in mice elevated circulating FGF19 levels and subsequently reduced high-fat diet-induced weight gain, increased metabolic rate and improved obesity-associated metabolic defects, including insulin resistance, liver steatosis and plasma lipid levels [15], [16]. Treatment of DIO mice or leptin-deficient *ob/ob* mice with bolus injections of recombinant FGF19 increased metabolic rate, increased weight loss, improved liver steatosis and enhanced body glucose utilization [16]. Importantly, we found that the magnitude of metabolic rate increase was similar between FGFR4 ASO-treated DIO mice and those in which plasma FGF19 was matched by a constant infusion to the level of FGF15 of the ASO-treated mice. The changes in metabolic rate and glucose metabolism observed in FGFR4 ASO-treated DIO mice mirror the changes in DIO mice associated with increased plasma FGF 19 as shown in the current studies or previous studies [15], [16]. Therefore, reduced adiposity and improved metabolic profile in DIO mice after antisense reduction of FGFR4 expression is mediated, at least in part, through the elevation of plasma FGF15 levels; and endogenously induced increase of plasma FGF15 levels after FGFR4 ASO treatment is as equally efficacious as the increase of plasma FGF19 levels either through pharmacological dosing or transgenic over-expression as reported previously [15], [16].

It is noteworthy that this is the first report to show that FGF15 circulates in plasma. Studies have well demonstrated that FGF19 circulates in plasma and plays important role, as a hormone, in energy metabolism and bile acids metabolism, and FGF15 is believed to be its ortholog in rodents [36]. However, due to lack of a good assay, circulating FGF15 has never been confirmed. Here, we developed a sensitive enzyme-linked immunosorbent assay for the determination of plasma FGF15 levels. The assay not only confirmed its presence in circulation but demonstrated its concentration in plasma well correlates with its expression in distal ileum and is regulated by bile acids in gut.

FGFR4 ASO treatment increased expression of hepatic Cyp7α and bile acid pool size as well as plasma bile acid levels. Bile acids, as a special class of signaling molecules, have recently been found to play an important role in energy metabolism and glucose homeostasis [37], [38]. Bile acids can bind to the cell surface receptor TGR5 that is expressed in a variety of tissues. Activation of TGR5 by bile acids in brown adipose tissue and muscle can in turn result in increased energy expenditure [37]. Feeding mice a high-fat diet supplemented with cholic acid increased energy expenditure and lowered BW [39], [40]. Thus, increased plasma bile acids after FGFR4 ASO treatment may also play some positive role in mediating FGFR4 ASO-induced anti-obesity effect. Further studies are warranted to determine the relative contribution of the increased plasma FGF15 levels versus bile acids in this regard, and to determine if there are any other factors that also mediate the FGFR4 ASO-induced anti-obesity effect.

Recent studies suggested that FGFR4 may play a broad role in glucose metabolism in addition to its involvement in regulation of bile acid synthesis [12], [13]. Expression of FGFR4 in liver was found to be decreased by fasting, increased by insulin, and reduced by streptozotocin-induced diabetes, implicating FGFR4 as a primary target of insulin regulation [12]. Huang et al found that FGFR4 knockout mice had different metabolic profile under different nutritional conditions [13]. The knockout mice showed increased fat mass, and increased plasma triglyceride, free fatty acid and cholesterol levels, and were glucose intolerant and insulin resistant when fed a normal diet. These phenotypes, however, were not different between the knockout mice and wild-type mice when fed a high-fat diet. Furthermore, the FGFR4 knockout mice showed improved liver steatosis when fed a high-fat diet. Unfortunately, no data on metabolic rate or plasma FGF15 levels in these FGFR4 knockout mice were reported [13], [41]. In the current studies, we found that DIO mice, after reduction of FGFR4 expression with a pharmacological approach, showed a different metabolic profile than that reported in FGFR4-deficient mice. This difference in metabolism could be due to many possible reasons, including different approaches (genetic approach versus pharmacological approach) used for the reduction of FGFR4 expression, the degree of FGFR4 reduction (global ablation in knockout mice versus specific peripheral reduction of 70–80% in ASO-treated mice) and the starting time of FGFR4 reduction (started from embryonic stage in knockout mice versus during adulthood with ASO treatment). Wu and colleagues found that although FGFR4 is essential for FGF15/19 to suppress Cyp7α, FGFR4 was not essential for FGF19 to improve glucose and lipid metabolism either in high-fat diet-fed mice or in leptin-deficient ob/ob mice [42]. The positive effects observed in the current studies on glycemia and energy metabolism associated with increased FGF15/19 despite reduction of FGFR4 expression with ASOs were consistent with their findings. Further studies are warranted to determine which specific receptor(s) is (are) required for FGF15/19 to play its positive roles in metabolism.

In summary, to investigate the potential metabolic effects of FGFR4 inhibition, DIO mice were treated under multiple therapeutic regimens with two different FGFR4 ASOs, which bind to separate regions of FGFR4 mRNA. Both FGFR4 ASOs markedly and specifically reduced liver FGFR4 expression. By increasing energy expenditure (coupled with decreasing lipogenesis), treatment with either ASO lowered BW, fat depot weight and whole body fat content. The improvement of adiposity was maintained during caloric restriction and, furthermore, the combination of FGFR4 ASO and rimonabant showed additive reduction in BW and adiposity. FGFR4 ASO treatment also improved insulin sensitivity, liver steatosis and plasma lipid levels. These data demonstrated that specific inhibition of FGFR4 could be a potential therapeutic approach for the treatment of obesity and related metabolic defects.

## References

[1] Hill JO (2006) Understanding and addressing the epidemic of obesity: an energy balance perspective. Endocr Rev 27: 750–761. 17122359 10.1210/er.2006-0032

[2] Saris WH (2001) Very-low-calorie diets and sustained weight loss. Obes Res 9 Suppl 4295S–301S. 11707557 10.1038/oby.2001.134

[3] Anderson JW, Konz EC, Frederich RC, Wood CL (2001) Long-term weight-loss maintenance: a meta-analysis of US studies. Am J Clin Nutr 74: 579–584. 11684524 10.1093/ajcn/74.5.579

[4] Leibel RL, Rosenbaum M, Hirsch J (1995) Changes in energy expenditure resulting from altered body weight. N Engl J Med 332: 621–628. 7632212 10.1056/NEJM199503093321001

[5] Schoeller DA (2008) Insights into energy balance from doubly labeled water. Int J Obes (Lond) 32 Suppl 7S72–75. 10.1038/ijo.2008.241 19136994

[6] Rosenbaum M, Leibel RL (2010) Adaptive thermogenesis in humans. Int J Obes (Lond) 34 Suppl 1S47–55. 20935667 10.1038/ijo.2010.184 PMC3673773

[7] Vetter ML, Faulconbridge LF, Webb VL, Wadden TA (2010) Behavioral and pharmacologic therapies for obesity. Nat Rev Endocrinol 6: 578–588. 20680034 10.1038/nrendo.2010.121 PMC3031864

[8] Padwal RS, Majumdar SR (2007) Drug treatments for obesity: orlistat, sibutramine, and rimonabant. Lancet 369: 71–77. 17208644 10.1016/S0140-6736(07)60033-6

[9] Inagaki T, Choi M, Moschetta A, Peng L, Cummins CL, et al (2005) Fibroblast growth factor 15 functions as an enterohepatic signal to regulate bile acid homeostasis. Cell Metab 2: 217–225. 16213224 10.1016/j.cmet.2005.09.001

[10] Holt JA, Luo G, Billin AN, Bisi J, McNeill YY, et al (2003) Definition of a novel growth factor-dependent signal cascade for the suppression of bile acid biosynthesis. Genes Dev 17: 1581–1591. 12815072 10.1101/gad.1083503 PMC196131

[11] Choi M, Moschetta A, Bookout AL, Peng L, Umetani M, et al (2006) Identification of a hormonal basis for gallbladder filling. Nat Med 12: 1253–1255. 17072310 10.1038/nm1501

[12] Shin DJ, Osborne TF (2009) FGF15/FGFR4 integrates growth factor signaling with hepatic bile acid metabolism and insulin action. J Biol Chem 284: 11110–11120. 19237543 10.1074/jbc.M808747200 PMC2670116

[13] Huang X, Yang C, Luo Y, Jin C, Wang F, et al (2007) FGFR4 prevents hyperlipidemia and insulin resistance but underlies high-fat diet induced fatty liver. Diabetes 56: 2501–2510. 17664243 10.2337/db07-0648

[14] Potthoff MJ, Boney-Montoya J, Choi M, He T, Sunny NE, et al (2011) FGF15/19 regulates hepatic glucose metabolism by inhibiting the CREB-PGC-1alpha pathway. Cell Metab 13: 729–738. 21641554 10.1016/j.cmet.2011.03.019 PMC3131185

[15] Tomlinson E, Fu L, John L, Hultgren B, Huang X, et al (2002) Transgenic mice expressing human fibroblast growth factor-19 display increased metabolic rate and decreased adiposity. Endocrinology 143: 1741–1747. 11956156 10.1210/endo.143.5.8850

[16] Fu L, John LM, Adams SH, Yu XX, Tomlinson E, et al (2004) Fibroblast growth factor 19 increases metabolic rate and reverses dietary and leptin-deficient diabetes. Endocrinology 145: 2594–2603. 14976145 10.1210/en.2003-1671

[17] Bhatnagar S, Damron HA, Hillgartner FB (2009) Fibroblast growth factor-19, a novel factor that inhibits hepatic fatty acid synthesis. J Biol Chem 284: 10023–10033. 19233843 10.1074/jbc.M808818200 PMC2665057

[18] Kir S, Beddow SA, Samuel VT, Miller P, Previs SF, et al (2011) FGF19 as a postprandial, insulin-independent activator of hepatic protein and glycogen synthesis. Science 331: 1621–1624. 21436455 10.1126/science.1198363 PMC3076083

[19] Yu XX, Murray SF, Pandey SK, Booten SL, Bao D, et al (2005) Antisense oligonucleotide reduction of DGAT2 expression improves hepatic steatosis and hyperlipidemia in obese mice. Hepatology 42: 362–371. 16001399 10.1002/hep.20783

[20] Pandey SK, Yu XX, Watts LM, Michael MD, Sloop KW, et al (2007) Reduction of low molecular weight protein-tyrosine phosphatase expression improves hyperglycemia and insulin sensitivity in obese mice. J Biol Chem 282: 14291–14299. 17353188 10.1074/jbc.M609626200

[21] Yu XX, Murray SF, Watts L, Booten SL, Tokorcheck J, et al (2008) Reduction of JNK1 expression with antisense oligonucleotide improves adiposity in obese mice. Am J Physiol Endocrinol Metab 295: E436–445. 18523126 10.1152/ajpendo.00629.2007

[22] Miyata M, Matsuda Y, Nomoto M, Takamatsu Y, Sato N, et al (2009) Cholesterol feeding prevents hepatic accumulation of bile acids in cholic acid-fed farnesoid X receptor (FXR)-null mice: FXR-independent suppression of intestinal bile acid absorption. Drug Metab Dispos 37: 338–344. 18988759 10.1124/dmd.108.022590 PMC2680521

[23] Savage DB, Choi CS, Samuel VT, Liu ZX, Zhang D, et al (2006) Reversal of diet-induced hepatic steatosis and hepatic insulin resistance by antisense oligonucleotide inhibitors of acetyl-CoA carboxylases 1 and 2. J Clin Invest 116: 817–824. 16485039 10.1172/JCI27300 PMC1366503

[24] Harrigan GG, Yates LA (2006) High-throughput screening, metabolomics and drug discovery. IDrugs 9: 188–192. 16523384

[25] Harrigan GG (2006) Metabolic profiling--IBC's inaugural meeting. Using metabolomics to accelerate drug discovery and development. 14–15 November 2005, Durham, NC, USA. IDrugs 9: 28–31. 16374729

[26] Yu XX, Drackley JK, Odle J (1997) Rates of mitochondrial and peroxisomal beta-oxidation of palmitate change during postnatal development and food deprivation in liver, kidney and heart of pigs. J Nutr 127: 1814–1821. 9278565 10.1093/jn/127.9.1814

[27] Choi CS, Savage DB, Kulkarni A, Yu XX, Liu ZX, et al (2007) Suppression of diacylglycerol acyltransferase-2 (DGAT2), but not DGAT1, with antisense oligonucleotides reverses diet-induced hepatic steatosis and insulin resistance. J Biol Chem 282: 22678–22688. 17526931 10.1074/jbc.M704213200

[28] Heal DJ, Gosden J, Smith SL (2012) What is the prognosis for new centrally-acting anti-obesity drugs? Neuropharmacology. 10.1016/j.neuropharm.2012.01.017 22313529

[29] Chakrabarti R (2009) Pharmacotherapy of obesity: emerging drugs and targets. Expert Opin Ther Targets 13: 195–207. 19236237 10.1517/14728220802637063

[30] Das SK, Chakrabarti R (2006) Antiobesity therapy: emerging drugs and targets. Curr Med Chem 13: 1429–1460. 16719787 10.2174/092986706776872880

[31] Maclean PS, Bergouignan A, Cornier MA, Jackman MR (2011) Biology's response to dieting: the impetus for weight regain. Am J Physiol Regul Integr Comp Physiol 301: R581–600. 21677272 10.1152/ajpregu.00755.2010 PMC3174765

[32] Samaha FF, Chou CM (2009) Blockade of the endocannabinoid system for the reduction of cardiometabolic risk factors. Obesity (Silver Spring) 17: 220–225. 19039319 10.1038/oby.2008.476

[33] Ravinet Trillou C, Arnone M, Delgorge C, Gonalons N, Keane P, et al (2003) Anti-obesity effect of SR141716, a CB1 receptor antagonist, in diet-induced obese mice. Am J Physiol Regul Integr Comp Physiol 284: R345–353. 12399252 10.1152/ajpregu.00545.2002

[34] Wiley JL, Burston JJ, Leggett DC, Alekseeva OO, Razdan RK, et al (2005) CB1 cannabinoid receptor-mediated modulation of food intake in mice. Br J Pharmacol 145: 293–300. 15778743 10.1038/sj.bjp.0706157 PMC1576140

[35] Christopoulou FD, Kiortsis DN (2011) An overview of the metabolic effects of rimonabant in randomized controlled trials: potential for other cannabinoid 1 receptor blockers in obesity. J Clin Pharm Ther 36: 10–18. 21198716 10.1111/j.1365-2710.2010.01164.x

[36] Jones SA (2012) Physiology of FGF15/19. Adv Exp Med Biol 728: 171–182. 22396169 10.1007/978-1-4614-0887-1_11

[37] Fiorucci S, Mencarelli A, Palladino G, Cipriani S (2009) Bile-acid-activated receptors: targeting TGR5 and farnesoid-X-receptor in lipid and glucose disorders. Trends Pharmacol Sci 30: 570–580. 19758712 10.1016/j.tips.2009.08.001

[38] Thomas C, Gioiello A, Noriega L, Strehle A, Oury J, et al (2009) TGR5-mediated bile acid sensing controls glucose homeostasis. Cell Metab 10: 167–177. 19723493 10.1016/j.cmet.2009.08.001 PMC2739652

[39] Watanabe M, Houten SM, Mataki C, Christoffolete MA, Kim BW, et al (2006) Bile acids induce energy expenditure by promoting intracellular thyroid hormone activation. Nature 439: 484–489. 16400329 10.1038/nature04330

[40] Watanabe M, Horai Y, Houten SM, Morimoto K, Sugizaki T, et al (2011) Lowering bile acid pool size with a synthetic farnesoid X receptor (FXR) agonist induces obesity and diabetes through reduced energy expenditure. J Biol Chem 286: 26913–26920. 21632533 10.1074/jbc.M111.248203 PMC3143650

[41] Yu C, Wang F, Kan M, Jin C, Jones RB, et al (2000) Elevated cholesterol metabolism and bile acid synthesis in mice lacking membrane tyrosine kinase receptor FGFR4. J Biol Chem 275: 15482–15489. 10809780 10.1074/jbc.275.20.15482

[42] Wu AL, Coulter S, Liddle C, Wong A, Eastham-Anderson J, et al (2011) FGF19 regulates cell proliferation, glucose and bile acid metabolism via FGFR4-dependent and independent pathways. PLoS One 6: e17868. 21437243 10.1371/journal.pone.0017868 PMC3060878

